# Survey of dermatophytes in stray dogs and cats with and without skin lesions in Puerto Rico and confirmed with MALDI-TOF MS

**DOI:** 10.1371/journal.pone.0257514

**Published:** 2021-09-24

**Authors:** Andrea Hernandez-Bures, Jason B. Pieper, Willie A. Bidot, Miranda O’Dell, William E. Sander, Carol W. Maddox

**Affiliations:** 1 Department of Veterinary Clinical Medicine, College of Veterinary Medicine, University of Illinois, Urbana, Illinois, United States of America; 2 Department of Veterinary Clinical Sciences, Iowa State University, Ames, Iowa, United States of America; 3 Office of Animal Resources, Western University of Health Sciences, Pomona, California, United States of America; 4 University of Illinois Veterinary Diagnostic Laboratory, College of Veterinary Medicine, University of Illinois, Urbana, Illinois, United State of America; 5 Department of Pathobiology, College of Veterinary Medicine, University of Illinois, Urbana, Illinois, United States of America; Fisheries and Oceans Canada, CANADA

## Abstract

Dermatophytosis is a common and highly contagious zoonotic skin disease in companion animals. This disease is a major concern in geographical areas that contain large numbers of stray animal populations. Numerous surveys on dermatophytosis among stray animal populations worldwide range between 27% to 50%. In recent years, the US territory of Puerto Rico was impacted by several natural disasters such as hurricanes, which has led to a large increase of abandonment cases and an increase in the stray animal population. Due to this, large low-cost spay/neuter clinics and trap-neuter-release programs have become a more common practice on the island. During these events, veterinary staff are exposed to multiple animals with no health history, and therefore, zoonotic diseases are of concern. The aim of this study was to provide information regarding the presence of dermatophyte species in symptomatic and asymptomatic stray dogs and cats in a region of Puerto Rico. Hair samples were collected from 99 stray animals with and without dermatological clinical signs. The hair samples were cultured on plates containing rapid sporulation medium and dermatophyte test medium. All cultures were evaluated microscopically to confirm the presence of dermatophytes. Then, all dermatophytes were further evaluated with MALDI-TOF MS to compare both diagnostic tests. A total of 19 animals (19%) were positive for dermatophyte growth. Of these animals, 18/19 were infected with *M*. *canis* and 1/19 with *Trichophyton* spp. Animals with clinical lesions were positive only 13.5% of the time compared to asymptomatic animals, who were positive in 36% of the sample population. All 19 dermatophytes (100%) diagnosed with microscopic evaluation were confirmed with MALDI-TOF MS. Our results indicate that there is a prevalence of 19% of dermatophytosis among the stray dog and cat population of the southeastern coast of the island.

## Introduction

Dermatophytosis is a common and highly contagious zoonotic skin disease in companion animals. It is caused by a superficial fungal infection that feeds off of keratinized tissues in both humans and animals [1, 2]. The most common fungal organisms that affect dogs and cats are *Microsporum canis*, *M*. *gypseum*, and *Trichophyton mentagrophytes* complex [2]. The prevalence of dermatophytes in companion animals has been reported all over the globe and their significance depends on geographical location and clinical and living conditions of the animals and humans [3].

Dermatophytosis becomes a major concern in geographical areas that contain large numbers of stray animal populations. In recent years, the US territory of Puerto Rico was impacted by several natural disasters such as hurricanes, which has led to a large increase of abandonment cases and an increase in the stray animal population. Due to a poor animal control system on the island, shelters were already over-crowded across the island before these events. There are numerous reports on the surveillance of dermatophytosis among stray animal populations in other countries which range between 27% to 50% regardless of clinical signs [4–8]. In most studies, the most commonly isolated dermatophyte is *M*. *canis* followed by *T*. *mentagrophytes* complex [4–7].

As an island with tropical weather year-round and a high prevalence of zoonotic pathogens such as leptospirosis [9, 10] and rabies [11], the stray animal population has become a public health concern. It is important to have an idea of the health status of the free-roaming stray animal population on the island to obtain information about pathogen and disease prevalence that might be silently affecting Puerto Rico. Thanks to aid provided through hurricane relief efforts, large low-cost spay/neuter clinics and trap-neuter-release (TNR) programs have become a more common practice on the island. These initiatives are a great way to engage the public, better control the stray population, and provide necessary preventative medicine [12]. Unfortunately, during these large events, veterinary staff are exposed to multiple animals with no health history, and therefore, zoonotic diseases are of concern.

Traditionally, the gold standard for diagnosing dermatophyte species has been by morphological analysis of specimens grown on fungal cultures [1, 13]. However, MALDI-TOF MS has become more popular for microorganism identification in the laboratory setting [14]. MALDI-TOF MS is a technique in which particles are ionized, separated according to their mass-to-charge ratio, and then measured by defining the time it takes for these ions to travel to a detector at the end of the time-of-flight tube. Then the results compared to a database of spectra from known organisms [15].

Compared with traditional morphology-based techniques, MALDI-TOF MS involves a rapid result turnaround time and can yield more accurate results without the need for highly trained staff [14, 16, 17]. Due to the increasing usage of MALDI-TOF MS in a clinical microbiology setting, we chose this modality for MS testing as it is clinically applicable.

To the authors’ knowledge, no reports have been published on the incidence or prevalence of dermatophytosis in stray animals in Puerto Rico. The aim of this study was to provide information regarding the presence of dermatophyte species in symptomatic and asymptomatic stray dogs and cats in a region of Puerto Rico. Additionally, we identify the dermatophytes using the traditional morphological analysis of isolates from fungal cultures and then confirm the species using MALDI-TOF MS.

## Materials and methods

### Institutional review and approval

This study was approved by the Institutional Animal Care and Use Committee at the University of Illinois and a memorandum of understanding was established with the non-profit organization hosting the event. All animals were managed routinely following the standard practices of the non-profit, with the only difference that hair samples were collected prior to surgery.

### Study population

Hair samples were collected from 99 stray animals (55 dogs and 44 cats) with and without dermatological clinical signs. Animals included in this study lived in both rural and urban areas and were trapped by local law enforcement of municipalities in the southeast of Puerto Rico, mainly the municipality of Guayama. The animals were trapped in cages and brought to a mobile style surgical site located in Guayama.

### Sample collection

All animals were anesthetized with a combination of TTDex [2.5mL of dexmedetomomidine (500mcg/mL) and 2.5mL of butorphanol tartarate (10mg/mL) added to a 5mL vial of unconstituted Telazol® (tiletamine and zolazepam 100mg/mL, Zoetis Inc, Parsippany, NJ, USA)] intra-muscularly. Animals were supplied with oxygen and if necessary for deeper anesthesia, isoflurane gas. Once the animal was completely anesthetized, the animal was brought to a pre-surgical table where a dermatological examination was performed, and hair samples were collected. Lesions were noted on the record (alopecia, crusts, scaling, pustules, papules, excoriations, erosions, ulcerations, and epidermal collarettes). The gross presence or absence of ectoparasites (fleas, ticks, and lice) was also recorded.

After dermatologic examination, hair samples were collected using the modified Mackenzie collection method with a sterile toothbrush by doing 20 brush strokes [2, 18]. The entire body of the animal was sampled starting from the head, followed by the neck, dorsum, trunk, ventrum, limbs and tail. After specimen collection, the toothbrushes were placed in wax paper bags (Reynolds Kitchens®, Richmond, VA) for transportation purposes.

After completion of sample collection, the animal went through the usual spay and neuter process, beginning with surgical preparation and then surgery. All animals were recovered safely after surgery.

### Fungal culture

The hair samples were cultured on plates containing two sections. Section I contained rapid sporulation medium and section II contained dermatophyte test medium (Derm-Duet™, Hardy Diagnostics, Santa Maria, CA). All samples were inoculated by gently pressing the toothbrush on medium surface (20 repetitions) [19] alternating between sections I and II at random order. The Petri dishes were incubated upside down in a room with normal laboratory lighting and at a constant temperature of 23˚C and examined daily for 21 days for evidence of growth [20]. If dermatophyte growth was detected via microscopic examination, the isolates were subcultured onto a Sabouraud’s dextrose agar (SDA) plate (Hardy Diagnostics, Santa Maria, CA). The isolates were monitored daily for macromorphological and micromorphological examination of colonies.

### Matrix-assisted laser desorption/ionization time-of-flight mass spectrometry (MALDI-TOF MS)

The MALDI-TOF MS analysis was conducted at the University of Illinois Veterinary Diagnostic Laboratory, College of Veterinary Medicine, University of Illinois using Bruker™ Biotyper Microflex LT, Bruker Daltonics, Bremen, Germany. It has a nitrogen laser with maximum frequency of 60 Hz, a minimum laser focus of 50 um, and 2,000 resolution in linear mode. It uses pulsed ionization with variable repetition rate and the upper mass limit is around 300 kDa. The optimum m/z ranges from 1000–3500 m/z.

### MALDI-TOF MS sample preparation

Most samples were prepared using the extended direct (ED) method. Samples that were not successfully identified with ED, underwent the filamentous fungi extraction (FE) method as per manufacturer’s manual (Bruker™ Biotyper **Microflex LT,** Bruker Daltonics, Bremen, Germany). For the ED method, all isolates were subcultured on SDA plates, and then were incubated upside down in a room with normal laboratory lighting and at a constant temperature of 23˚C. Microscopic identification of the desired colonies was followed by the ED method of MALDI-TOF MS. Hyphal growth was selected from fresh colonies using a toothpick to make a uniform, thin film on a spot of the steel MALDI target (MSP 96 target polished steel BB; Bruker Daltonics, Bremen, Germany) and allowed to air dry. Once dried, 1μl of 70% formic acid was added to the spot and allowed to air dry. Next, 1μl of matrix (HCCA (Matrix α-cyano-4-hydroxycinnamic acid, #255344, Bruker Daltonics, Bremen, Germany) was added to the smear and allowed to air dry. The template was placed in the instrument and the vacuum established prior to scanning. A bacterial test strain (BTS) control spot was used to validate the laser and detector and each sample spot was evaluated using “smart acquisition” which accumulates spectra and as a consensus mass spectrum profile (MSP) emerges, it completes that identification scan, so the number of shots varies. The MSPs were compared to the Bruker™ MBT Compass version 4.1.100 data base containing 577 reference fungal MSPs to find a best match. Using a 0 to 3 scoring system, scores less that 1.69 are unreliable, 1.70–1.99 are low confidence identities while scores > 2.0 are acceptable high confidence identities for most speciation. If an acceptable ID was not obtained by ED, the FE method was used as described below.

For the FE method, active growth from fresh SDA cultures was inoculated into 8-mL tubes of Sabouraud broth (BD™ BBL Mycoflask). The tubes were placed on a rotator at 23˚C until sufficient biological material was observed. The cultures were then left to stand for approximately 10 min. Up to 1.5 mL filamentous fungal sediment was collected from the bottom of the tube and transferred to a 1.5-mL Eppendorf tube. The samples were centrifuged (Beckman Coulter Microfuge 16, Beckman Coulter Inc., Indiana, USA) for 2 min at 12,470 x g. After the removal of the supernatant, 1 mL of HPLC-grade water was added to the pellet, and the sample was vortexed for 1 minute. The centrifugation was repeated, and then 300 μl of HPLC-grade water was added to the pellet. Subsequently, 900 μl of 100% ethanol was added to the mixture. The sample was centrifuged at 12,470 x g for 2 minutes. The supernatant was discarded, and ethanol was completely removed following an additional centrifugation step.

In the next stage, the pellet was dried at 23°C. Then 10–100 μl of 70% formic acid were added (depending on the size of the pellet), an equal volume of acetonitrile was added, the samples were mixed well then centrifuged again at 12,470 x g for 2 minutes. The supernatant (1 μl) was transferred to a MALDI target and allowed to dry at 23°C. Finally, the sample was overlaid with 1 μl of saturated α-cyano-4-hydroxycinnamic acid solution (HCCA matrix; Bruker Daltonics, Bremen, Germany). The matrix and sample were allowed to air dry at 23°C. Following drying of the target plate, the sample was loaded for MALDI-TOF MS analysis as above.

### Statistical analysis

Since all data was categorical, 2-way contingency tables were used to calculate Chi-square values or Fishers Exact test values (when any expected count was less than 5). Additionally, odds ratios (OR) were determined. Statistical significance was set at *p*≤ 0.05.

## Results

**Fig 1:**
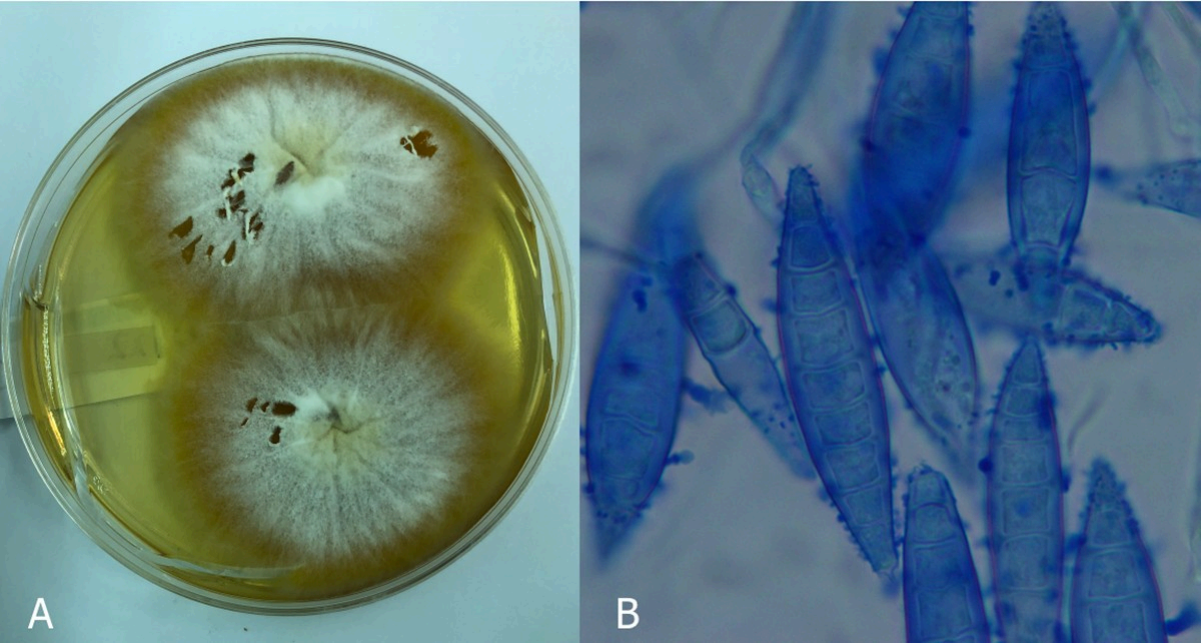
*Microsporum canis* dermatophyte. (A) Macroscopic identification of *M*. *canis* dermatophyte. Grown at 23˚C on SDA with a filamentous form, raised center and flat periphery. Has white to cream color. (B) Microscopic identification of *M*. *canis* dermatophyte. Numerous spindle-shaped macroconidia with 5–10 cells.

**Fig 2:**
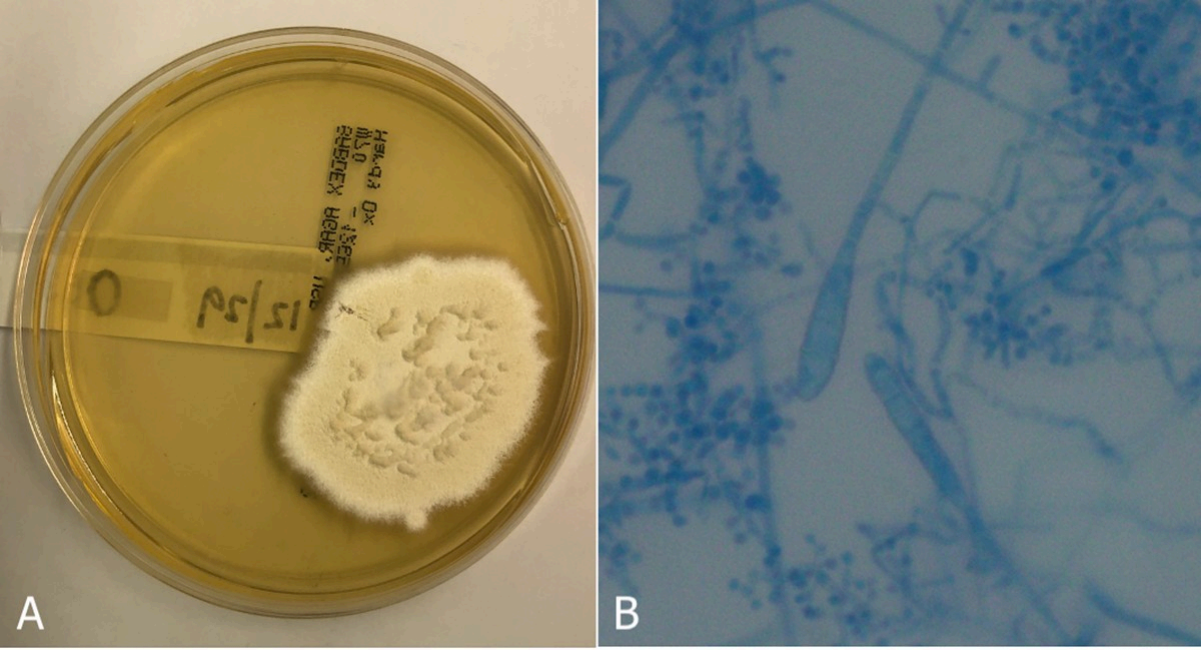
*Trichophyton spp*. dermatophyte. (A) Macroscopic identification of *Trichophyton spp*. dermatophyte. Grown at 23˚C on SDA with an irregular form, flat, and undulate margins. Texture appears granular and is white in color. (B) Microscopic identification of *Trichophyton spp*. dermatophyte. Two club-shaped macroconidia and multiple spherical microconidia in clusters.

Samples were collected from 55 stray dogs and 44 stray cats from the southeast region of the island. Of the total animals sampled (n = 99), 37 were males and 62 were females. The animal ages were estimated and categorized as juveniles (n = 38) and adults (n = 61). A total of 19 animals (19%) were positive for dermatophyte growth. Of these animals, 18/19 were infected with *M*. *canis* and 1/19 with *Trichophyton* spp (Figs 1 and 2). Prevalence rates of dermatophytes were 29.5% of feline animals compared to 10.9% of canine animals, which was statistically significant [OR (feline) 3.43 (1.18–9.95)]. Positive cultures were observed in 34.2% of juvenile animals compared to 9.8% of adult animals, which was statistically significant [OR (juvenile) 3.48 (1.22–9.93)]. Animals with clinical lesions were positive only 13.5% of the time compared to asymptomatic animals were positive in 36% of the sample population, which was statistically significant [OR (clinical lesions) 0.246 (0.08–0.72)]. The most prevalent lesions noted on symptomatic animals were scaling (100.0%) and alopecia with scaling (33.3%). For hair color, there was a higher prevalence of 62.5% in black and black predominant cats compared to 22.2% of other colored cats, which was statistically significant [OR (black cats) 5.83 (1.14–29.85); p = 0.037]. Epidemiological data of the population and prevalence of dermatophytes are summarized in Table 1. Additional characteristics such as hair types, other hair colors, presence of ectoparasites, and types of lesions are listed as supporting material.

**Table 1:** Epidemiological data.

Variable	Categories	Number of subjects	%	Number and percentage of animals positive for dermatophytes	*P* value
**Sex**	Female	62	62.6	11/62 (17.7%)	0.793
	Male	37	37.4	8/37 (21.6%)	
**Age**	Young	38	38.4	13/38 (34.2%)	0.006
	Adult	61	61.6	6/61 (9.8%)	
**Species**	Dogs	55	55.5	6/55 (10.9%)	0.037
	Cats	44	44.4	13/44 (29.5%)	
**Skin lesion**	Yes	74	74.4	10/74 (13.5%)	0.018
	No	25	25.3	9/25 (36.0%)	
**Ectoparasites**	Yes	28	28.2	4/28 (14.2%)	0.621
	No	71	71.1	15/71 (21.1%)	
**Positivity to dermatophytes**	Yes	19	19.2	-	
	No	80	80.8	-	
**Dermatophyte species identified**	*M*. *Canis*	18	18.2	-	
	*Trichophyton spp*.	1	1.0	-	

Epidemiological data of the population and prevalence of dermatophytes in relation to the epidemiological data in a population of 99 stray dogs and cats. Statistical significance was set at *p*≤0.05.

All cultures were evaluated microscopically to determine the presence of dermatophytes. All organisms were identified as best as possible based on culture and microscopic characteristics. All dermatophytes were further evaluated with MALDI-TOF MS to compare both diagnostics tests. A MALDI-TOF MS score of greater than 2.0 was considered highly accurate to the species level. All 19 dermatophytes (100%) diagnosed with microscopic evaluation were confirmed with MALDI-TOF MS (Table 2).

**Table 2:** Dermatophytes with their MALDI-TOF MS scores.

Animal ID	Species	Organism	MALDI-TOFMS Score
050	Feline	*M*. *canis*	2.46
059	Feline	*M*. *canis*	2.37
060	Feline	*M*. *canis*	2.15
061	Feline	*M*. *canis*	2.00
062	Feline	*M*. *canis*	2.00
063	Feline	*M*. *canis*	2.20
066	Feline	*M*. *canis*	2.03
067	Feline	*M*. *canis*	2.02
053	Canine	*Trichophyton* spp.	2.13
065	Canine	*M*. *canis*	2.24
072	Feline	*M*. *canis*	2.05
073	Feline	*M*. *canis*	2.06
074	Feline	*M*. *canis*	2.03
075	Feline	*M*. *canis*	2.13
087	Feline	*M*. *canis*	2.24
081	Canine	*M*. *canis*	2.40
084	Canine	*M*. *canis*	2.19
085	Canine	*M*. *canis*	2.15
090	Canine	*M*. *canis*	2.05

Nineteen animals diagnosed with dermatophytes via microscopic diagnosis and confirmed with MALDI-TOF MS.

## Discussion

This study was performed to evaluate the presence of dermatophytosis on the skin and hair coats of stray dogs and cats in Puerto Rico. There was a 19% prevalence of dermatophytosis in all animals, where 29.5% of all cats and 10.9% of all dogs were affected. Most importantly, the percentage of asymptomatic animals in the population that tested positive were 36%, whereas symptomatic animals that tested positive were 13.5%. The dermatophyte species most commonly noted was *M*. *canis*, which is not surprising as the majority of the affected animals were felines who are known to be asymptomatic carriers [1]. This poses a major risk for contamination of environments, and transmission to other animals and humans, which can be a concern in high volume spay/neuter clinics. To help decrease the likelihood of transmission between other animals and staff members in a large spay/neuter clinic or in a TNR event, several safety measures should be considered.

Of the affected animals, there was a higher prevalence of positive cultures in cats compared to dogs and more juveniles (particularly cats) were affected than adults. Cats would be expected to have a higher rate of positive cultures since *M*. *canis* is the dermatophyte most isolated from both affected and asymptomatic cats [1–3]. It is also more commonly reported that young age and old age are risk factors for acquiring dermatophytosis [1]. In our study group, over half of the cats sampled were juveniles and no senior cats were sampled. In addition to young age being a risk factor, the sample size discrepancy between species can also influence the higher number of affected juvenile felines compared to affected juvenile canines. Future survey studies should include a larger sample size and a broader range of age groups to evaluate if this is a persistent trend.

We did not notice any breed predilections among both dogs and cats. This could have been due to the small sample size and the fact that most cats seen in the clinic were domestic short hair (DSH). There was also no relationship found between animals having ectoparasites and dermatophytosis prevalence. It has been previously reported that microtrauma resulting from pruritus induced by ectoparasites can predispose animals to dermatophytosis [4, 21]. Compared to other hair coat colors, there was a statistically significant higher prevalence of black-haired cats with dermatophytosis. There are no reports in either human or veterinary medicine indicating a higher prevalence of dermatophytosis in pigmented skin or hair. Additional studies should look into the correlation of tissue pigmentation and prevalence of dermatophytosis as other diseases have shown a correlation.

Dermatophytosis has been noted as one of the principal public health concerns worldwide, and close contact with animals, poor hygiene, and environmental circumstances are the most influential factors in the transmission of this disease [8, 22]. The prevalence of dermatophytosis tends to be higher in tropical and subtropical countries since warmer and humid environments favor the growth of these fungi [23]. However, there are no reports indicating whether there are increases in the amount of dermatophytosis cases after natural disasters such as tropical storms and hurricanes. We suspect that an increase in rainfall and flooding, which leads to high humidity levels, can lead to an increase in numbers of dermatophyte infections. More survey studies are warranted to evaluate the prevalence of dermatophytosis in humans and animals in these humid regions.

We were able to accurately confirm with MALDI-TOF MS the diagnosis of all 19 dermatophytes that were identified microscopically. Previous human studies have evaluated the accuracy of identifying dermatophytes with in clinic DTM cultures compared to MALDI-TOF MS identification [14, 16, 17, 24, 25]. MALDI-TOF MS conveys several benefits over conventional morphology-based techniques. The first one being that MALDI-TOF MS involves a rapid result turnaround time [14]. Compared to morphological examination, MALDI-TOF MS can take less than a week to obtain a diagnosis compared to a DTM culture, which requires 21 days for completion. Additionally, to have an accurate morphological diagnosis, macroconidia are required, which can be time sensitive and take up to 21 days. For MALDI-TOF MS, the growth stage of the dermatophyte and the culture medium used has no effect on the species identification [25]. Another advantage of MALDI-TOF MS is that it does not require labor-intensive and experienced training like it does for dermatophyte identification in the clinical setting [14, 17]. However, a reference data library is required with MALDI-TOF MS identification [14].

Our study’s major limitation would be the small group of animals used for the study. Our goal was to be able to identify the prevalence of dermatophyte cases in the real-world setting of a TNR event on the island, which in most situations depend on volunteers that are able to successfully trap the animals. For future studies, larger groups of animals would ideally be used to have a better representation of the prevalence depending on age, species, and breeds.

To the authors’ knowledge, this is the first dermatophytosis survey study performed in stray dogs and cats of Puerto Rico. Our results indicate that there is a prevalence of 19% of dermatophytosis among the stray dog and cat population of the southeastern coast of the island. Additional survey studies are needed to establish trends in other parts of the island and determine if the environment (e.g., rural vs urban) and natural disasters (e.g., hurricanes) are influencing factors.

## Supporting information

S1 Table Signalment data of the population and dermatophyte prevalence. Signalment data of the population and prevalence of dermatophytes in relation to the signalment for each risk factor considered in 99 stray dogs and cats with and without clinical signs in the southeast region of Puerto Rico.
(DOCX)

S2 Table Phenotypic data and prevalence of dermatophytes. Phenotypic data of the population and prevalence of dermatophytes in relation to the phenotype for each risk factor considered in 99 stray dogs and cats with and without clinical signs in the southeast region of Puerto Rico.
(DOCX)

S3 Table Ectoparasites of the population and dermatophyte prevalence. Gross ectoparasite findings of the population and prevalence of dermatophytes in relation to gross ectoparasites for each risk factor considered in 99 stray dogs and cats with and without clinical signs in the southeast region of Puerto Rico. **One positive dog had both fleas and ticks.
(DOCX)

S4 Table Lesions and prevalence of dermatophytes. Lesions noted on the physical exams of the population and prevalence of dermatophytes in relation to these lesions for each risk factor considered in 99 stray dogs and cats with and without clinical signs in the southeast region of Puerto Rico. **Some animals had multiple lesions.
(DOCX)
