# Supplementary material for: Survey of dermatophytes in stray dogs and cats with and without skin lesions in Puerto Rico and confirmed with MALDI-TOF MS
Source: PLoS One. 2021 Sep 24;16(9):e0257514. doi: 10.1371/journal.pone.0257514 (PMC8462699; doi:10.1371/journal.pone.0257514)
Supplement: S1 Table — Signalment data of the population and prevalence of dermatophytes in relation to the signalment for each risk factor considered in 99 stray dogs and cats with and without clinical signs in the southeast region of Puerto Rico. (DOCX) [file pone.0257514.s001.docx]

**S1 Table. Signalment Data of the Population and Dermatophyte Prevalence.**

| **Variable** | **Positives** | **Total Population** | **%** |
| --- | --- | --- | --- |
| **Species** | 19 | 99 | 19.2 |
| Canine | 6 | 55 | 10.9 |
| Feline | 13 | 44 | 29.5 |
|  |  |  |  |
| **Age** | 19 | 99 | 19.2 |
| Juveniles | 13 | 38 | 34.2 |
| Juvenile dog | 3 | 15 | 20.0 |
| Juvenile cat | 10 | 23 | 43.5 |
| Adults | 6 | 61 | 9.8 |
| Adult dog | 3 | 41 | 7.3 |
| Adult cat | 3 | 20 | 15.0 |
|  |  |  |  |
| **Sex** | 19 | 99 | 19.2 |
| Male | 8 | 37 | 21.6 |
| Female | 11 | 62 | 17.7 |
|  |  |  |  |
| **Breed** | 19 | 99 | 19.2 |
| Chihuahua Mix | 0 | 1 | 0.0 |
| Chow Mix | 0 | 1 | 0.0 |
| Domestic medium hair | 2 | 7 | 28.6 |
| Domestic short hair | 11 | 36 | 30.6 |
| Labrador Mix | 0 | 1 | 0.0 |
| Maltese Mix | 0 | 1 | 0.0 |
| Mixed Breed | 3 | 30 | 10.0 |
| Pitbull | 0 | 4 | 0.0 |
| Rottweiler Mix | 0 | 1 | 0.0 |
| Shepherd Mix | 1 | 5 | 20.0 |
| Siamese Mix | 0 | 1 | 0.0 |
| Terrier Mix | 2 | 11 | 18.2 |

Signalment data of the population and prevalence of dermatophytes in relation to the signalment for each risk factor considered in 99 stray dogs and cats with and without clinical signs in the southeast region of Puerto Rico.
