# Supplementary material for: Survey of dermatophytes in stray dogs and cats with and without skin lesions in Puerto Rico and confirmed with MALDI-TOF MS
Source: PLoS One. 2021 Sep 24;16(9):e0257514. doi: 10.1371/journal.pone.0257514 (PMC8462699; doi:10.1371/journal.pone.0257514)
Supplement: S2 Table — Phenotypic data of the population and prevalence of dermatophytes in relation to the phenotype for each risk factor considered in 99 stray dogs and cats with and without clinical signs in the southeast region of Puerto Rico. (DOCX) [file pone.0257514.s002.docx]

**S2 Table. Phenotypic Data and Prevalence of Dermatophytes.**

| **Variable** | **Positives** | **Total Population** | **%** |
| --- | --- | --- | --- |
| **Hair Length** | 19 | 99 | 19.2 |
| Short | 16 | 82 | 19.5 |
| Dog Short | 5 | 46 | 10.9 |
| Cat Short | 11 | 36 | 30.6 |
| Medium | 2 | 11 | 18.2 |
| Dog Medium | 0 | 3 | 0.0 |
| Cat Medium | 2 | 8 | 25.0 |
| Long | 1 | 6 | 16.7 |
| Dog Long | 1 | 6 | 16.7 |
| Cat Long | 0 | 0 | 0.0 |
|  |  |  |  |
| **Hair Color** | 19 | 99 | 19.2 |
| Black only cat | 5 | 7 | 71.4 |
| Black only dog | 0 | 3 | 0.0 |
| Black predominant cat | 0 | 1 | 0.0 |
| Black predominant dog | 1 | 19 | 5.3 |
| Brindle dog | 0 | 1 | 0.0 |
| Brown only dog | 0 | 4 | 0.0 |
| Brown only cat | 2 | 5 | 40.0 |
| Brown predominant cat | 0 | 1 | 0.0 |
| Brown predominant dog | 1 | 4 | 25.0 |
| Calico | 0 | 1 | 0.0 |
| Cream Dog | 2 | 4 | 50.0 |
| Grey only cat | 1 | 1 | 100.0 |
| Grey only dog | 0 | 1 | 0.0 |
| Grey predominant cat | 0 | 1 | 0.0 |
| Grey predominant dog | 0 | 1 | 0.0 |
| Orange only cat | 2 | 9 | 22.2 |
| Orange predominant cat | 0 | 1 | 0.0 |
| Tan only dog | 1 | 6 | 16.7 |
| Tan predominant dog | 0 | 3 | 0.0 |
| Tortoise Shell | 1 | 2 | 50.0 |
| White only dog | 0 | 2 | 0.0 |
| White only cat | 0 | 1 | 0.0 |
| White predominant Dog | 1 | 7 | 14.3 |
| White predominant Cat | 2 | 14 | 14.3 |

Phenotypic data of the population and prevalence of dermatophytes in relation to the phenotype for each risk factor considered in 99 stray dogs and cats with and without clinical signs in the southeast region of Puerto Rico.
