# Supplementary material for: Survey of dermatophytes in stray dogs and cats with and without skin lesions in Puerto Rico and confirmed with MALDI-TOF MS
Source: PLoS One. 2021 Sep 24;16(9):e0257514. doi: 10.1371/journal.pone.0257514 (PMC8462699; doi:10.1371/journal.pone.0257514)
Supplement: S3 Table — Gross ectoparasite findings of the population and prevalence of dermatophytes in relation to gross ectoparasites for each risk factor considered in 99 stray dogs and cats with and without clinical signs in the southeast region of Puerto Rico. **One positive dog had both fleas and ticks. (DOCX) [file pone.0257514.s003.docx]

**S3 Table. Ectoparasites of the Population and Dermatophyte Prevalence.**

| **Variable** | **Positives** | **Total Population** | **%** |
| --- | --- | --- | --- |
| **Ectoparasites** | 19 | 99 | 19.2 |
| Flea | 4 | 22 | 18.2 |
| Cats with fleas | 2 | 8 | 25.0 |
| Dogs with fleas | 2 | 14 | 14.2 |
| Lice | 0 | 4 | 0.0 |
| Cats with lice | 0 | 0 | 0.0 |
| Dogs with lice | 0 | 4 | 0.0 |
| Ticks | 1 | 3 | 33.3 |
| Cats with ticks | 0 | 0 | 0.0 |
| Dogs with ticks | 1 | 3 | 33.3 |
| None | 15 | 71 | 21.1 |
| Cats with no ectoparasites | 12 | 38 | 31.6 |
| Dogs with no ectoparasites | 3 | 33 | 9.1 |

Gross ectoparasite findings of the population and prevalence of dermatophytes in relation to gross ectoparasites for each risk factor considered in 99 stray dogs and cats with and without clinical signs in the southeast region of Puerto Rico. **One positive dog had both fleas and ticks.
