# Supplementary material for: Survey of dermatophytes in stray dogs and cats with and without skin lesions in Puerto Rico and confirmed with MALDI-TOF MS
Source: PLoS One. 2021 Sep 24;16(9):e0257514. doi: 10.1371/journal.pone.0257514 (PMC8462699; doi:10.1371/journal.pone.0257514)
Supplement: S4 Table — Lesions noted on the physical exams of the population and prevalence of dermatophytes in relation to these lesions for each risk factor considered in 99 stray dogs and cats with and without clinical signs in the southeast region of Puerto Rico. **Some animals had multiple lesions. (DOCX) [file pone.0257514.s004.docx]

**S4 Table. Lesions and Prevalence of Dermatophytes.**

| **Variable** | **Positives** | **Total Population** | **%** |
| --- | --- | --- | --- |
| **Lesions** | 19 | 99 | 19.2 |
| Alopecia cat | 2 | 14 | 14.3 |
| Alopecia dog | 5 | 48 | 10.4 |
| Alopecia, Crusts cat | 1 | 3 | 33.3 |
| Alopecia, crusts dog | 2 | 26 | 7.7 |
| Alopecia, Excor Cat | 0 | 1 | 0.0 |
| Alopecia, Excor dog | 2 | 13 | 15.4 |
| Alopecia, Scaling cat | 0 | 1 | 0.0 |
| Alopecia, scaling dog | 2 | 11 | 18.2 |
| Crusts cat | 2 | 7 | 28.6 |
| Crusts dog | 2 | 21 | 9.5 |
| Excoriations cat | 0 | 2 | 0.0 |
| Excoriations dog | 1 | 12 | 8.3 |
| No significant findings (NSF) | 9 | 25 | 36.0 |
| NSF adult dog | 0 | 1 | 0.0 |
| NSF cat | 9 | 24 | 37.5 |
| NSF adult cat | 2 | 9 | 22.2 |
| NSF juvenile cat | 7 | 15 | 46.7 |
| Papules cat | 0 | 1 | 0.0 |
| Papules dog | 0 | 4 | 0.0 |
| Scaling cat | 1 | 1 | 100.0 |
| Scaling dog | 2 | 9 | 22.2 |
| Ulcerations dog | 1 | 7 | 14.2 |
| Ulcerations cat | 0 | 0 | 0.0 |
| Aural hematoma dog | 0 | 1 | 0.0 |
| Clinical lesions | 10 | 74 | 13.5 |
| Clinical Lesions dog | 6 | 55 | 10.9 |
| Clinical lesions cat | 4 | 19 | 21.0 |

Lesions noted on the physical exams of the population and prevalence of dermatophytes in relation to these lesions for each risk factor considered in 99 stray dogs and cats with and without clinical signs in the southeast region of Puerto Rico. **Some animals had multiple lesions.
